# Supplementary material for: Spherulitic and rotational crystal growth of Quartz thin films
Source: Sci Rep. 2021 Jul 21;11:14888. doi: 10.1038/s41598-021-94147-y (PMC8295350; doi:10.1038/s41598-021-94147-y)
Supplement: Supplementary file 1 — Supplementary Information. [file 41598_2021_94147_MOESM1_ESM.docx]

**Supplementary information for:**

Spherulitic and rotational crystal growth of Quartz thin films

by: Nick R. Lutjes, Silang Zhou, Jordi Antoja-Lleonart, Beatriz Noheda, Václav Ocelík

Zernike Institute for Advanced Materials, University of Groningen, Nijenborgh 4, 9747 AG Groningen, The Netherlands

1. **Trigonal α-Quartz symmetry**

Figure S1a) summarizes the trigonal symmetry of α-Quartz by showing some important crystal planes on the pole figure. Stereographic projections of the poles of some crystal planes are shown. Four digit Miller-Bravais indices are used and crystal *c* axis is orthogonal to the figure plane. Some commonly used notations^1^ are also introduced: crystal directions [0001] and <$11\bar{2}0$> are denoted as [c] and <a> as the main crystallographic axes. The poles of planes (0001), {$10\bar{1}0$}, {$10\bar{1}1$}, {$01\bar{1}1$}, {$10\bar{1}2$} and {$01\bar{1}2$} are denoted as (c), {m}, {r}, {z}, {π} and {π’}, respectively. Crystal facets that correspond to these lattice planes are shown in Fig. S1b), using colors matching the pole figure.


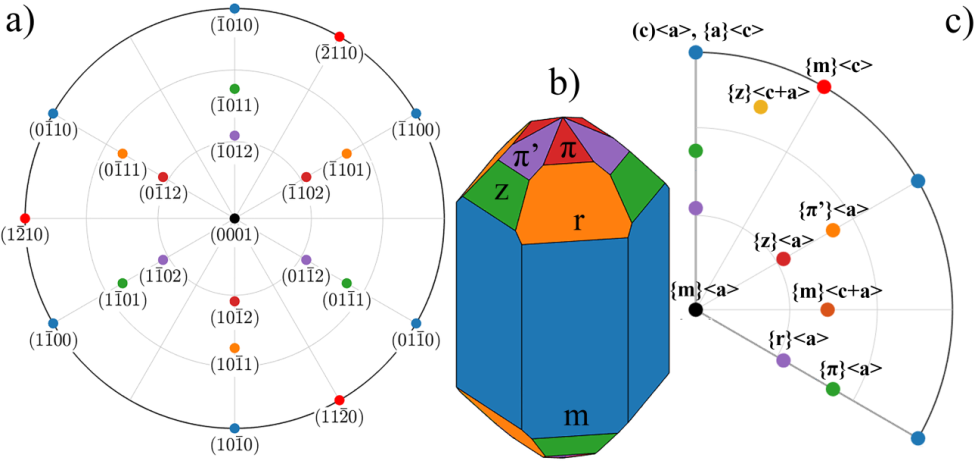


**Fig. S1:** Quartz crystallography:
a) Quartz crystal pole figure showing normal to the important crystallographic planes.
b) A shape of Quartz crystal with important plane’s facets and their conventional abbreviations. The color of the facets indicates the corresponding normal axes in a) or c).
c) Inverse pole figure of Quartz crystal with highlighted rotation axes of common slip systems summarized in Table S1.

1. **Spherulite fiber individualization procedure**

Fibers individualization process we may characterized in following steps:

a) In the first step the misorientation boundaries were constructed, using a threshold of misorientation angle of 3º. This value has been selected keeping in mind two facts: i) the angular resolution in determination of misorientation between two points on the same EBSD map is about 0.2º and ii) the quartz lattice inside spherulites rotates with a rate of about 1º/µm. For experimental scanning steps between 0.1 – 1 µm, the misorientation angle value of 3º is an adequate choice to include also boundaries with small misorientation. These misorientation boundaries separate the individual fibers inside quartz spherulites studied in our work.

b) Primary fibers are then defined as areas that contain (or are adjacent to) the spherulite nucleation center, which means there is no low angle boundary that separates points inside primary fibers and the spherulite nucleation center. The spherulite nucleation center is approximately determined: i) visually (for non-epitaxial nucleated spherulites) on the EBSD map as a geometrical center of the whole object with the help of the misorientation boundaries lines, which converge to the center, or ii) as the pixel which shows its crystal orientation closest to that of the substrate. Generally, the area containing the spherulite nucleation center splits into few primary fibers, each characterized by its main growing direction out of the spherulite center.

c) Secondary fibers are then simply all other areas separated from primary fibers by a grain boundary inside the corresponding spherulite.

1. **α-Quartz dislocations slip systems**

Common dislocations slip systems in α-Quartz crystal are summarized in Table S1. Figure S1c) shows the corresponding directions of crystal rotation axis in the Inverse pole figure.

**Table S1:** Summarized common slip systems in Quartz crystal (SPN: Slip Plane Normal, SD: Slip Direction) and corresponding crystal Rotation axis according ^2–4^.

| Slip system | | | | Rotation axis | |
| --- | --- | --- | --- | --- | --- |
| SPN | SD | SPN | SD |  |  |
| Symbol | | (hkil) | [UVTW] | symbol | [UVTW] |
| (c) | <±a> | (0001) | <$11\bar{2}0$> | {m} | [$1\bar{1}00$] |
| {a} | [c] | {$11\bar{2}0$} | [0001] | {m} | [$\bar{1}100$] |
| {m} | <±a> | {$10\bar{1}0$} | <$1\bar{2}10$> | (c) | [0001] |
| {z} | <±a> | {$01\bar{1}1$} | <$2\bar{11}0$> | {π} | [$01\bar{1}\bar{2}$] |
| {r} | <±a> | {$10\bar{1}1$} | <$1\bar{2}10$> | {π’} | [$\bar{1}012$] |
| {m} | [c] | {$10\bar{1}0$} | [0001] | {a} | [$\bar{1}2\bar{1}0$] |
| {m} | <c+a> | {$10\bar{1}0$} | <$\bar{1}2\bar{1}3$> |  | [$\bar{0.418} 0.836 \bar{0.418} \bar{1.000}$] |
| {z} | <c+a> | {$01\bar{1}1$} | <$\bar{1}\bar{1}23$> |  | [$\bar{1.000} 0.687 0.313 \bar{0.374}$] |
| {π} | <±a> | {$10\bar{1}2$} | <$1\bar{2}10$> | {z} | [$10\bar{1}\bar{1}$] |
| {π’} | <±a> | {$1\bar{1}02$} | <$11\bar{2}0$> | {r} | [$\bar{1}101$] |

**References:**

1. Kilian, R. & Heilbronner, R. Analysis of crystallographic preferred orientations of experimentally deformed Black Hills Quartzite. *Solid Earth* **8**, 1095–1117 (2017).

2. Lloyd, G. E. Microstructural evolution in a mylonitic quartz simple shear zone: the significant roles of dauphine twinning and misorientation. *Geological Society, London, Special Publications* **224**, 39–61 (2004).

3. Neumann, B. Texture development of recrystallised quartz polycrystals unravelled by orientation and misorientation characteristics. *Journal of Structural Geology* **22**, 1695–1711 (2000).

4. Linker, M. F., Kirby, S. H., Ord, A. & Christie, J. M. Effects of compression direction on the plasticity and rheology of hydrolytically weakened synthetic quartz crystals at atmospheric pressure. *J. Geophys. Res.* **89**, 4241–4255 (1984).
